# Caregiver Willingness to Vaccinate Their Children against COVID-19 after Adult Vaccine Approval

**DOI:** 10.3390/ijerph181910224

**Published:** 2021-09-28

**Authors:** Ran D. Goldman, Danna Krupik, Samina Ali, Ahmed Mater, Jeanine E. Hall, Jeffrey N. Bone, Graham C. Thompson, Kenneth Yen, Mark A. Griffiths, Adi Klein, Eileen J. Klein, Julie C. Brown, Rakesh D. Mistry, Renana Gelernter, on behalf of the International COVID-19 Parental Attitude Study (COVIPAS) Group

**Affiliations:** 1 The Pediatric Research in Emergency Therapeutics (PRETx) Program, Department of Pediatrics, Division of Emergency Medicine, BC Children’s Hospital Research Institute, University of British Columbia, Vancouver, BC V6H 3N1, Canada; 2 Ziv Medical Center, Pediatric Emergency Unit, Azrieli Faculty of Medicine, Bar-Ilan University, Safed 5290002, Israel; dannak@ziv.gov.il; 3 Departments of Pediatrics, Faculty of Medicine & Dentistry, Women and Children’s Health Research Institute, University of Alberta, Edmonton, AB T6G 2R3, Canada; sali@ualberta.ca; 4 Pediatric Emergency Medicine, Jim Pattison Children’s Hospital, University of Saskatchewan, Saskatoon, SK S7N 5B5, Canada; mater999@me.com; 5 Division of Emergency and Transport Medicine, Children’s Hospital Los Angeles, USC Keck School of Medicine, Los Angeles, CA 90033, USA; jehall@chla.usc.edu; 6 Biostatistical Lead, Research Informatics, BC Children’s Hospital Research Institute, Vancouver, BC V5Z 4H4, Canada; jeffrey.bone@cw.bc.ca; 7 Pediatrics and Emergency Medicine, Alberta Children’s Hospital, University of Calgary, Calgary, AB T2N 1N4, Canada; graham.thompson@albertahealthservices.ca; 8 Division of Pediatric Emergency Medicine, Department of Pediatrics, University of Texas Southwestern Children’s Health, Dallas, TX 75235, USA; ken.yen@utsouthwestern.edu; 9 Division of Pediatric Emergency Medicine, Children’s Healthcare of Atlanta, Emory School of Medicine, Atlanta, GA 30322, USA; mark.anthony.griffiths@emory.edu; 10 Hillel Yaffe Medical Center, Department of Pediatrics, Hadera and Technion Faculty of Medicine, Haifa 38100, Israel; adi@hy.health.gov.il; 11 Seattle Children’s Hospital, University of Washington School of Medicine, Seattle, WA 98195, USA; eileen.klein@seattlechildrens.org (E.J.K.); Julie.brown@seattlechildrens.org (J.C.B.); 12 Department of Pediatrics, Section of Emergency Medicine, University of Colorado Denver, Aurora, CO 80204, USA; rakesh.mistry@childrenscolorado.org; 13 Shamir Medical Center, Pediatric Emergency Medicine Unit, Sackler Faculty of Medicine, Tel Aviv University, Tel Aviv 6997801, Israel; grenana@gmail.com

**Keywords:** vaccine hesitancy, parental attitudes, COVID-19

## Abstract

Vaccines against COVID-19 are likely to be approved for children under 12 years in the near future. Understanding vaccine hesitancy in parents is essential for reaching herd immunity. A cross-sectional survey of caregivers in 12 emergency departments (ED) was undertaken in the U.S., Canada, and Israel. We compared reported willingness to vaccinate children against COVID-19 with an initial survey and post-adult COVID-19 vaccine approval. Multivariable logistic regression models were performed for all children and for those <12 years. A total of 1728 and 1041 surveys were completed in phases 1 and 2, respectively. Fewer caregivers planned to vaccinate against COVID-19 in phase 2 (64.5% and 59.7%, respectively; *p* = 0.002). The most significant positive predictor of willingness to vaccinate against COVID-19 was if the child was vaccinated per recommended local schedules. Fewer caregivers plan to vaccinate their children against COVID-19, despite vaccine approval for adults, compared to what was reported at the peak of the pandemic. Older caregivers who fully vaccinated their children were more likely to adopt vaccinating children. This study can inform target strategy design to implement adherence to a vaccination campaign.

## 1. Introduction

Vaccine hesitancy, defined as a delay in the acceptance or refusal of vaccines despite the availability of vaccine services [1,2], has increased over recent years [3,4,5,6] and was recognized as one of the top ten threats to global health in 2019 [7]. A 2019 national survey from the United States (U.S.) found that approximately one out of four parents reported serious concerns towards vaccinating their children [8] and parental vaccine hesitancy in regard to their children may hamper efforts to curtail the COVID-19 pandemic [9]. In March 2020, the Center for Disease Control and Prevention’s (CDC) National Immunization Survey data revealed that more than 30% of U.S. children between the ages of 19 and 35 months were not following the recommended early childhood immunization schedule [10]. The complexity of vaccine hesitancy is derived from over 70 independent variables [11], predominantly psychological barriers (e.g., perceived risk, usefulness, and social benefit), contextual barriers (e.g., access to health care services), and lifestyle (e.g., smoking, drinking, and physical activity) [12]. 

As vaccines for children under 12 years are in late stages of clinical trials, there is importance in understanding parental attitudes and preferences in regard to accepting future COVID-19 vaccines for their children. This information can help public heath officers design specific campaigns to enhance vaccine acceptance and improve herd immunity. Previous studies have focused on understanding and addressing individual and community vaccine hesitancy [4,5,6,13]. Concerns about vaccine hesitancy have been further heightened during the COVID-19 pandemic, at a time when reaching herd immunity is essential to public health and mitigation of widespread infection. At the end of 2020, during the height of the COVID-19 pandemic, the U.S. Food and Drug Administration (FDA) provided emergency approval for COVID-19 vaccines, and Americans grew confident in their safety and effectiveness, with 60% stating they definitely or probably would get vaccinated [14]. Yet, when it came to vaccinating children, a poll of over 1200 parents in May 2021 revealed that just half (53%) planned to vaccinate eventually, and only a quarter of parents (26%) reported they would vaccinate ‘right away’ [15]. Our study team recently reported that among 1541 parents visiting 16 emergency departments (EDs) in six countries during the peak of the COVID-19 pandemic, 65% said they would vaccinate their children [16]. Another study conducted in England found that 48.2% parents of young infants indicated they would vaccinate their children [17]. However, lower COVID-19 vaccine acceptance rates in children were reported in other recent studies (33.9–36.3%) [18,19].

In May 2021, a COVID-19 vaccine was first approved for ages 12–15 years by Health Canada [20] and subsequently by the FDA in the U.S. Our study focused on the experience of parents who may have received the vaccine themselves or learned about the vaccine experience of others either from conversations or the media, prior to approval for use in children or adolescents. The objective of this follow-up study was to determine how COVID-19 vaccine approval for adults was associated with caregiver likelihood to vaccinate their children.

## 2. Materials and Methods

### 2.1. Sample and Procedures

This cross-sectional study was part of the COVID-19 Parental Attitude Study (COVIPAS) of caregivers presenting for emergency care for their children during the era of COVID-19. Subjects included all caregivers who arrived at one of 12 pediatric emergency departments (EDs) in the U.S. (Denver, Los Angeles, Dallas, Seattle, Atlanta), Canada (Vancouver, Saskatoon, Edmonton, Calgary) and Israel (Zerifin, Hedera, Safed). Caregivers were recruited using posters placed in waiting areas and patient rooms, as well as direct approach by team members. For infectious control purposes, caregivers used their own smartphones to scan a QR code to access the survey by logging into a secured online platform based on the REDCap metadata-driven platform (Vanderbilt University). Once a caregiver selected their study site, consent for participation was implied. This study was approved by each site’s local Institutional Review Board (IRB). 

The initial phase 1 was conducted during the peak of the pandemic (March–May 2020) and a revised survey, after a COVID-19 vaccine became available for adults through emergency authorization in all three countries (phase 2), was conducted from December 2020 to March 2021. The revised survey was available in English, French, and Hebrew. Only one caregiver completed the survey per visit, and due to restrictions to visitation in most sites, only one caregiver was in the room with each child.

### 2.2. Measures

The study-specific questionnaire was developed to include questions regarding demographic characteristics, information regarding the ED visit, and caregiver attitudes about vaccinating against COVID-19. The survey objective was to compare caregivers’ perspectives and actions before and after a vaccine was available for adults. We have previously described the development and validation of the original survey [16]. The main outcome measure in the study was the rate of parents who plan to vaccinate their children 12 years and younger once a vaccine becomes available. We also asked parents about their level of concern about them or their child having COVID-19, as well as losing work during the pandemic or their children losing school days. 

### 2.3. Data Analysis

Descriptive statistics and frequencies were used to describe all variables. Phase 1 and phase 2 were compared using the Mann–Whitney tests for comparing non-normal continuous variables, independent t-tests for comparing normally distributed continuous variables, and Chi-square or Fisher’s exact tests for comparing categorical variables, with a significance level of 0.05. We then fit multivariable logistic regression models stratified by phase to compare relevant a priori identified predictors of caregiver intent to vaccinate against COVID-19. Results from these models were summarized with adjusted odds ratios and 95% confidence intervals. Significance for the multivariable models was set at 0.05. Similar analyses were conducted in a subgroup of parents whose child being seen in the ED was less than 12 years of age. All analyses were conducted with R version 4.0.3 (R Core Team, Vienna, Austria).

## 3. Results

A total of 2769 surveys were completed with 1728 in phase 1 (pre adult vaccine approval) and 1041 in phase 2 (post vaccine approval 2020–2021). There were 1499 (54.1%) from Canada, 934 (33.7%) from the USA and 336 (12.1%) from Israel.

The study population and comparison between the population surveyed in phase 1 and phase 2 are presented in Table 1. Mean child age was 7.75 ± 5.33 years, 2003 (72.4%) were under 12 years of age, and 1352 (49%) were female. Eighty-nine percent (*n* = 2455) of parents reported their children were fully vaccinated according to the local pediatric schedule, and less than 2% (*n* = 51) were against vaccinations in children. Almost 75% of the surveys were completed by mothers (*n* = 2064), and the mean age of respondents was 38.7 years (SD 8.0).

Three percent (*n* = 82) of respondents had a household member who was confirmed to have been infected with COVID-19 and 200 (7.4%) children had a prior exposure to someone sick with COVID-19. Upon arrival to the ED, 732 (26.4%) had symptoms that could have been caused by COVID-19 (e.g., fever, respiratory symptoms).

In phase 2, there were significantly more children exposed to COVID-19 at home, tested for the viral illness, and found at one point in time to be positive for COVID-19. Although there were significantly fewer children with COVID-19-related symptoms upon presentation in phase 2, caregivers were more concerned either they or their children were sick with COVID-19 at the time of presentation to the ED, compared to phase 1. All other demographic characteristics of children between phases were similar (Table 1).

Overall, 1736 (62.7%) caregivers planned to vaccinate their children against COVID-19, and among parents of children under 12 years of age, 1223/2003 (61.1%) planned to vaccinate their children. Less caregivers planned to vaccinate their children against COVID-19 in phase 2 (1115; 64.5%), compared to phase 1 (621; 59.7%) (*p* = 0.002). For caregivers of children under 12 years, 808/1270 (63.6%) said they will vaccinate their children in phase 1, and 415/733 (56.6%) in phase 2 (*p* = 0.002). Demographic characteristics and key parameters in the survey were similar among those parents willing to vaccinate young children in both phases (Table 2). 

The most significant positive predictor of likelihood of a caregiver to report willingness to vaccinate their child against COVID-19 was their child being vaccinated per the recommended schedule. This finding was consistent across the entire cohort of caregivers, as well as for those with children under 12 years old. 

When a multivariable analysis was conducted for the likelihood of caregivers vaccinating their children against COVID-19 once a vaccine is available for them (Table 3), in phase 1, mothers were much less likely to vaccinate. Caregivers that were older, those worried the child had COVID-19 and those caregivers of children with up-to-date vaccinations were more likely to vaccinate for COVID-19. In phase 2, caregiver age and child’s immunization status remained predictors of willingness to vaccinate against COVID-19. Concern about losing future work was also positive predictor, while previous lost income was a negative predictor (Table 3).

When the multivariable analysis was conducted only including caregivers of children under 12 years of age (Table 4), we found in phase 1 that mothers and other caregivers were less likely to vaccinate, while older caregivers, those worried their children had COVID-19, and current up-to-date vaccinate status, were significantly associated with a plan to vaccinate their children. In phase 2, caregiver age and child’s immunization status remained predictors of willingness to vaccinate against COVID-19.

## 4. Discussion

Vaccinating children against COVID-19 is critical as a public health strategy, since they contribute to the spread of disease to higher risk adult populations [21] and in order to reduce the incidence of COVID-associated multisystem inflammatory system in children (MIS-C) [22]. 

When a modified survey was delivered in EDs seven months apart, demographics were very similar for those surveyed pre- and post-approval of a COVID-19 vaccine for adults. Fewer caregivers planned to vaccinate their children against COVID-19 in the post-approval phase. Caregivers of fully immunized children and older caregivers were most likely to immunize their children against COVID-19. In the larger group of caregivers, lost income due to the pandemic was a deterrent from vaccinating; however, those concerned about future lost wages were more likely to vaccinate. The latter two factors were not as important among caregivers of younger children.

Not surprisingly, we found more families were exposed to, or sick with, COVID-19 during the second phase. Caregivers were also more worried about the child or the caregiver having COVID-19 when arriving at the ED. 

In Bologna, Italy, during December 2020 and January 2021, an online survey reported that 60.4% of over 5000 parents were inclined to vaccinate, similar to findings in our cohort, and 29.6% were considering the opportunity [23]. Vaccine hesitancy (9.9%) was higher among caregivers who were mothers of children aged 6–10 years, younger than 29 years old, had lower educational level, relied on information found on the web/social media, and disliked mandatory vaccination policies [23]. In a recent online survey in the U.S. with 427 participants, 44% planned to vaccinate children against COVID-19 once the vaccine becomes available to them [24], reflecting similar findings as in our cohort after the vaccine was approved in adults. However, 75% of children were reported to have chronic conditions, a much higher rate than in the general population, or the cohort of families completing our international study. Among parents of young infants and toddlers (18 month or younger) in the U.K., 48.2% reported “Definitely” and 40.9% “Unsure but leaning towards yes” for vaccinating their children [17], which reflects a much higher rate of likelihood to vaccinate than we found. In one online survey of factory workers in Shenzhen, China, from September 2020, a higher rate (72.6% (764/1052)) of parents was willing to vaccinate their children for COVID-19. Family member’s support and feeling the child will take up vaccination were associated with higher parental acceptability [25]. Among almost 5000 parents within the school public health network of the same region, hesitancy was 27.3% and parents with psychological distress were more likely to be hesitant about vaccinating their children [26]. A self-administered online survey in Turkey at the same time as our phase 2 revealed much lower willingness to vaccinate children (36.3%) than in our survey, and in comparison to the rate of parents planning to get vaccinated themselves (59.9%). Of interest, willingness increased to 83.9% if mortality associated with COVID-19 in children increased following a mutation [19]. In another survey from a Children’s Hospital in Turkey, 66% of parents were reluctant to receive foreign COVID-19 vaccines, and 37% were reluctant to receive domestic COVID-19 vaccines [18]. These reports, along with our international sample, suggest significant geographic and time-dependent variability in willingness to vaccinate children, and the multitude of factors that may affect parents in deciding on vaccinating against COVID-19. 

Unfortunately, the ongoing pandemic is not associated with a shift towards vaccinating children; instead, parents are more hesitant, a source of great concern to the healthcare and scientific communities. The initial experience with safe adult COVID-19 vaccination in the countries participating in this study was not sufficiently reassuring for parents, and they did not report greater willingness to administer vaccines to their children, compared to pre-adult vaccine approval. This supports findings in a recent systematic review and meta-analysis on adults of 28 nationally representative samples (*n* = 58,656) from 13 countries during June–October 2020 [27], revealing a decline in willingness to vaccinate against COVID-19. It is possible that some parents felt reassured when hearing that children have a mild COVID-19 illness. Furthermore, at the time of this study, more information on COVID-19 presentation and course was available for parents through media and public health reports, compared to the initial period of the pandemic. Similar to our findings among parents, the meta-analysis reported females, younger, and of lower income or education level were consistently associated with limited willingness to get vaccinated [27].

Unlike our prior findings during the peak of the pandemic [16], desire to expedite vaccine research [28], and other reports on potential vaccine uptake [18,27], we found more balanced gender-dependent decision making on vaccination in the second phase of this study, and mothers were as likely as fathers to vaccinate (although confidence intervals overlapped between phases). This finding may be associated with the somewhat lower rate of mothers completing the second phase survey (76% and 72% in the first and second phase, respectively), and that parents were slightly older. It is possible that early in the pandemic (phase 1), mothers, who are usually responsible for health care decision making at the household, took children more often to visit the ED. This may have changed as the pandemic continued (phase 2). We are uncertain why older parents brought their children in the second phase. Those findings are in contrast to a widening gap in attitudes between fathers and mothers found in the U.S. where a quarter of mothers said they are “extremely unlikely” to vaccinate their children [29]. If concerns about safety of the vaccine [24] affect caregiver decision making, potential association of one COVID-19 vaccine to myocarditis in adolescents [30] may have further broadened this gap and public health organizations should find channels of communication directly to alleviate concerns expressed by caregivers. These may include advertising on social media channels to subgroups of the population, or through particular school systems in regions with a known high rate of vaccine hesitancy. 

Of interest, those already affected by pandemic-related lost income prior to entering the study did not seem to be interested in vaccinating their children, while those reporting more fear of future lost work favored vaccination. Perhaps parents who are worried about imminent or potential lost work, which frequently they have little control over, would like their children to get vaccinated as an activity they can control and, this way, to reduce the need to decide on vaccination at a later date while adding burden on the family. Among parents of young children (<12 years), economic factors were not significantly different between the two phases. More research is needed to explore these attitudes and age-related differences, likely by means of qualitative methodology, in order to understand parental priorities around health-related and economic-related factors. Vaccine hesitancy is an important factor in the decision to vaccinate children during this pandemic, and our study suggest that over 40% of caregivers need to be convinced to vaccinate their young children; addressing this will be an important part of a worldwide effort to reach herd immunity and achieve a post-pandemic world. Overcoming COVID-19 vaccine hesitancy among adults for their own vaccination may not be transferred to their interest in vaccination of their children, as we have shown at the peak of the pandemic [31]. Uncovering specific reasons for COVID-19 vaccine hesitancy is an urgent task and will help regional public health agencies ensure parents receive the answers to questions they have when considering available vaccines. Furthermore, the relationship between parents and health care providers affects their vaccine decision making [11], and communication strategies by providers are essential during the COVID-19 ongoing pandemic. Approaching families with older parents who are already convinced that a child needs to be fully vaccinated may result in earlier gains in vaccinating children, especially those under 12 years.

Our study has several limitations. First, as with many surveys, it is unlikely to be a representative sample of families visiting the EDs or the entire population in these three countries and, therefore, we cannot rule out response or selection biases. We also did not collect information on how many parents were already vaccinated at the time they responded to the survey in phase 2 (vaccines for adults were available under emergency authorization). Furthermore, as data on adverse events of vaccines continue to be published in the media, parents may have different perspectives than during the time our survey took place. Finally, rate of vaccination in different regions participating in the study may have affected parental attitudes.

## 5. Conclusions

In summary, 44% of caregivers do not plan to vaccinate their children against COVID-19, a higher rate than was seen in a similar cohort before vaccines for adults were approved. Older caregivers who fully vaccinated their children are more likely to vaccinate their 12–18 years old children now that the vaccine has been authorized for emergency use for this age group, and for younger children (under 12 years old) once it is available. These data could help in designing target strategies to implement adherence to a vaccination campaign. Health care providers can harness these findings to identify vaccine hesitancy among parents in their practice and communicate the importance of vaccinating young children, even before vaccines are available for this age group.

## Figures and Tables

**Table 1:** Characteristics of the survey population during the first phase (March–May 2020) and second phase (December 2020–March 2021). SD = Standard Deviation.

	Surveys (2769)	Total Population	Phase 1 (*n* = 1728)	Phase 2 (*n* = 1041)	*p*-Value
Child					
Child’s mean age in years (SD)	2768	7.75 (5.33)	7.62 (5.25)	7.98 (5.45)	0.088
Child Under 12 Years of Age	2768	2003 (72.4%)	1270 (73.5%)	733 (70.4%)	0.076
Child’s gender female	2762	1352 (49.0%)	861 (50.0%)	491 (47.2%)	0.006
Child has chronic illness	2768	399 (14.4%)	238 (13.8%)	161 (15.5%)	0.243
Child uses chronic medication	2768	493 (17.8%)	298 (17.3%)	195 (18.7%)	0.351
Child’s immunizations up to date	2748	2455 (89.3%)	1518 (88.7%)	937 (90.4%)	0.143
Child tested negative for COVID-19	2707	469 (17.3%)	136 (8.06%)	333 (32.7%)	<0.001
Someone at home was sick with COVID-19	2715	82 (3.02%)	16 (0.95%)	66 (6.43%)	<0.001
Someone at home had exposure to COVID-19	2711	200 (7.38%)	61 (3.61%)	139 (13.6%)	<0.001
Child had symptoms that may be attributed to COVID-19 on arrival to ED	2769	732 (26.4%)	506 (29.3%)	226 (21.7%)	<0.001
Caregiver					
Completed by mother	2769	2064 (74.5%)	1314 (76.0%)	750 (72.0%)	0.006
Caregiver’s age	2718	38.7 (7.98)	38.4 (8.09)	39.3 (7.77)	0.004
Caregiver has higher than high school education	2709	2159 (79.7%)	1328 (78.7%)	831 (81.3%)	0.115
Level of concern about child having COVID-19 (Likert Scale 0–10)	2667	1.99 (3.12)	1.78 (2.96)	2.36 (3.34)	<0.001
Level of concern about caregiver having COVID-19 (Likert Scale 0–10)	2649	1.78 (2.90)	1.60 (2.74)	2.10 (3.12)	<0.001
Level of concern about losing work (Likert Scale 0–10)	2623	2.93 (3.63)	2.76 (3.61)	3.21 (3.66)	0.003
Level of concern about child losing school (Likert Scale 0–10)	2612	2.94 (3.61)	2.80 (3.61)	3.18 (3.61)	0.009
Caregiver believes in social distancing as a good public health measure	2702	2493 (92.3%)	1583 (92.3%)	910 (92.2%)	0.981
Caregiver had lost income due to COVID-19	2694	1099 (40.8%)	718 (41.9%)	381 (38.8%)	0.120

**Table 2:** Parents of children 12 years and younger who will vaccinate their children by phase of study.

Summary of Those Planning to Vaccine Children under 12 Years	Entire Cohort (*n* = 2003)	Phase 1 Will Vaccinate (*n* = 1270)	Phase 2 Will Vaccinate (*n* = 733)	*p*-Value
Will vaccinate	1223 (61.1%)	808 (63.6%)	415(56.6%)	0.002
Child’s age in Years (SD) *	5.13 (3.62)	5.22 (3.62)	5.5 (3.65)	0.20
Child’s immunization up-to-date	1745 (87.8%)	731 (91.0%)	388 (93.5%)	0.17
Father completed the survey	470 (23.5%)	206 (25.5%)	115 (27.7%)	0.59
Mother completed the survey	1493 (74.5%)	588 (72.8%)	291 (70.1%)	
Other completed the survey	40 (2.00%)	14 (1.73%)	9 (2.17%)	
Caregiver’s age (Years) **	36.7 (7.14)	37.2 (7.41)	38.2 (6.63)	0.02
Parent has higher than high school education	1588 (80.8%)	651 (82.2%)	351 (86.5%)	0.07
Level of concern about child having COVID-19 (Likert scale 0–10)	2.11 (3.18)	2.11 (3.13)	2.75 (3.48)	0.002
Level of concern about losing work (Likert scale 0–10)	2.88 (3.62)	2.74 (3.51)	3.46 (3.66)	0.001
Parent had lost income due to COVID-19	806 (41.6%)	330 (41.3%)	144 (36.9%)	0.17

* For one month increase. ** For one year increase.

**Table 3:** Predictors of all caregivers’ willingness to vaccinate their children against COVID-19 identified by multivariable logistic regression analysis in first phase (March–May 2020) and second phase (December 2020–March 2021).

	Phase 1			Phase 2		
	Odds Ratio	95% CI	*p*-Value	Odds Ratio	95% CI	*p*-Value
Child’s age in years (SD) *	0.999	(0.997–1)	0.326	1.00	(0.999–1)	0.361
Child’s immunization up-to-date	2.54	(1.84–3.53)	<0.001	2.74	(1.70–4.51)	<0.001
Who completed the survey						
Father (Reference)						
Mother	0.557	(0.415–0.742)	<0.001	1.01	(0.726–1.40)	0.954
Other	0.748	(0.385–1.51)	0.403	1.31	(0.506–3.59)	0.588
Caregiver’s age (years) **	1.03	(1.01–1.05)	<0.001	1.04	(1.02–1.07)	<0.001
Parent has higher than high school education	1.24	(0.934–1.64)	0.136	1.11	(0.748–1.64)	0.606
Level of concern about child having COVID-19 (Likert scale 0–10)	1.09	(1.04–1.13)	<0.001	1.01	(0.967–1.06)	0.642
Level of concern about losing work (Likert Scale 0–10)	1.00	(0.969–1.03)	0.991	1.05	(1.01–1.09)	0.026
Parent had lost income due to COVID-19	0.852	(0.681–1.07)	0.163	0.73	(0.545–0.977)	0.034

* For one month increase. ** For one year increase.

**Table 4:** Predictors of willingness to vaccinate against COVID-19 among caregivers of children under 12 years, identified by multivariable logistic regression analysis in first phase (March–May 2020) and second phase (December 2020–March 2021).

	Phase 1			Phase 2		
	Odds Ratio	95% CI	*p*-Value	Odds Ratio	95% CI	*p*-Value
Child’s age in years (SD) *	0.999	(0.995–1)	0.393	1	(0.997–1.01)	0.392
Child’s immunization up-to-date	2.67	(1.87–3.83)	<0.001	1.97	(1.12–3.51)	0.020
Who completed the survey						
Father (Reference)						
Mother	0.522	(0.372–0.726)	<0.001	0.954	(0.647–1.4)	0.810
Other	0.335	(0.138–0.824)	0.015	2.16	(0.555–10.9)	0.298
Caregiver’s age (years) **	1.03	(1.01–1.05)	0.006	1.04	(1.01–1.08)	0.007
Parent has higher than high school education	1.23	(0.877–1.72)	0.230	1.43	(0.882–2.34)	0.146
Level of concern about child having COVID-19 (Likert scale 0–10)	1.1	(1.05–1.15)	<0.001	1.04	(0.989–1.1)	0.125
Level of concern about losing work (Likert Scale 0–10)	0.996	(0.96–1.03)	0.831	1.05	(0.998–1.1)	0.059
Parent had lost income due to COVID-19	0.917	(0.708–1.19)	0.510	0.725	(0.512–1.03)	0.070

* For one month increase. ** For one year increase.

## Data Availability

Data will be made available upon request.
